# Risk of acute kidney injury after contrast-enhanced computerized tomography: a systematic review and meta-analysis of 21 propensity score–matched cohort studies

**DOI:** 10.1007/s00330-022-08916-y

**Published:** 2022-06-21

**Authors:** Mikal Obed, Maria Magdalena Gabriel, Eva Dumann, Clara Vollmer Barbosa, Karin Weißenborn, Bernhard Magnus Wilhelm Schmidt

**Affiliations:** 1 Department of Nephrology and Hypertension, Hannover Medical School, Carl-Neuberg-Straße 1, 30625 Hannover, Germany; 2 Department of Neurology, Hannover Medical School, Carl-Neuberg-Straße 1, 30625 Hannover, Germany

**Keywords:** Acute kidney injury, Contrast media, Computed tomography, Glomerular filtration rate, Propensity score matching

## Abstract

**Objectives:**

Intravenous application of contrast media is part of a wide spectrum of diagnostic procedures for better imaging quality. Clinical avoidance of contrast-enhanced imaging is an ever-present quandary in patients with impaired kidney function. The objective of this study was to estimate the risk for acute kidney injury (AKI), dialysis and mortality among patients undergoing contrast-enhanced CT compared to propensity score–matched controls (i.e. contrast-unenhanced CT). Selected cohort studies featured high-risk patients with advanced kidney disease and critical illness.

**Methods:**

This review was designed to conform to the Preferred Reporting Items in Systematic Reviews and Meta-Analysis (PRISMA) guidelines. PubMed was searched from August 2021 to November 2021 for all-language articles without date restriction. A random-effects model (DerSimonian and Laird method) was used for meta-analysis.

**Results:**

Twenty-one articles were included, comprising data of 169,455 patients. The overall risk of AKI was similar in the contrast-enhanced and unenhanced groups (OR: 0.97 [95% CI: 0.85; 1.11], *p* = 0.64), regardless of baseline renal function and underlying disease. Substantial heterogeneity was detected (*I*^2^ = 90%, *p* ≤ 0.0001). Multivariable logistic regression identified hypertension (*p* = 0.03) and estimated glomerular filtration rate (eGFR) ≤ 30 mL/min/1.73 m^2^ (*p* = 0.0001) as factors associated with greater risk of post-contrast AKI.

**Conclusions:**

Based on propensity score–matched pairs obtained from 21 cohort studies, we found no evidence for increased risk for AKI, dialysis or mortality after contrast-enhanced CT among patients with eGFR ≥ 45 mL/min/1.73 m^2^. In congruence with the emerging evidence in the literature, caution should be exercised in patients with hypertension and eGFR ≤ 30 mL/min/1.73 m^2^.

**Key Points:**

• *The application of contrast media for medical imaging is not associated with higher odds for AKI, induction of renal replacement therapy, or mortality. Many comorbidities traditionally associated with greater risk for acute kidney injury do not appear to predispose for renal decline after contrast media exposure.*

• *Underlying hypertension and eGFR less than or equal to 30 mL/min/1.73 m*^*2*^ *seem to predispose for post-contrast acute kidney injury.*

• *Propensity score matching cannot account for unmeasured influences on AKI incidence, which needs to be addressed in the interpretation of results.*

**Supplementary Information:**

The online version contains supplementary material available at 10.1007/s00330-022-08916-y.

## Introduction

Contrast-induced nephropathy (CIN), also known as contrast-associated acute kidney injury (CA-AKI) or contrast-induced acute kidney injury (CI-AKI), is defined as a rapid decline of renal function within days following intravascular exposure to contrast media (CM) [1, 2]. CA-AKI is traditionally suggested to be a leading cause of hospital-acquired acute kidney injury (AKI), presenting 12% of all cases [3]. The absolute and relative definitions of CA-AKI are diverse. Most commonly, it is characterized by an absolute increase in serum creatinine (SCr) levels of ≥ 0.3 mg/dL or ≥ 0.5 mg/dL from baseline. It can also be defined as a relative SCr increase of more than 25% of baseline or 1.5 times baseline within 1–3 or 4–5 days after intravenous or intra-arterial CM application [4–6]. By definition, no factors other than previous CM exposure can provide sufficient explanation for the renal decline [7, 8]. Post-contrast AKI is strongly associated with short- and long-standing adverse and potentially irreversible outcomes [9, 10].

Contrast-enhanced computed tomography (CT) is an indispensable component of medical imaging. Although low- and iso-osmolar contrast agents are generally considered safe, their intravenous administration for greater imaging quality and diagnostic accuracy has been assumed one of the most frequent causes of AKI in clinical practice [7].

However, numerous cohort studies have challenged this historic belief by using the propensity score (PS) to match CM–exposed subjects with unexposed controls. Propensity score matching (PSM) is an analytical approach to estimate the weight of CM exposure on the incidence of AKI [11]. Applying logistic regression, patients are matched according to similar distributions of baseline characteristics [11, 12]. Studies applying the PS have revealed equal rates of AKI in matched cohorts (i.e. CM exposed and unexposed), pointing out the underestimated role of underlying comorbidities in the development of AKI. Thus, it is suggested that the dreaded deterioration of kidney function following contrast-enhanced imaging might have been falsely attributed to CM rather than the susceptibility of particular patient collectives.

The objective of this systematic review and meta-analysis was to determine the risks of acute nephropathy in patients undergoing contrast-enhanced CT compared with demographically similar controls undergoing contrast-unenhanced CT. As secondary outcomes of interest, we evaluated the risks of dialysis and mortality in patients with CM–enhanced and unenhanced CT.

## Materials and methods

### Protocol and registration

The protocol for this systematic review was registered at the International Prospective Register of Systematic Reviews (PROSPERO) under the identification number 197088, and was accepted on September 6, 2020.

### Data sources and search

Two investigators (M.O. and B.M.W.S.) reviewed all English-language publications in Cochrane Library, PubMed and MEDLINE using the search terms “propensity score AND contrast media“ and “(AKI OR nephropathy) AND (iodinated contrast OR contrast media OR CT OR angiography) AND propensity score” with no date restrictions. Data extraction was performed until November 2021, using a predefined set of inclusion and exclusion criteria. In case of differing results between both researchers, a third (K.W.) and a fourth (E.D.) investigator reviewed and adjudicated the results.

### Eligibility criteria

All studies reporting on the effects of CM exposure on AKI incidence using a propensity score–matching model were considered for inclusion. Eligible studies required two arms: one group of patients undergoing CM–enhanced CT and a control group undergoing unenhanced CT. Studies were included for meta-analysis if AKI was defined by Acute Kidney Injury Network (AKIN); Kidney Disease: Improving Global Outcomes (KDIGO); Risk, Injury, Failure, Loss of kidney function, and End-stage Kidney Disease (RIFLE); or contrast-induced nephropathy (CIN) criteria [13–16]. The presence of SCr measurements or glomerular filtration rates (GFRs) before and after CT scans was required for inclusion. No age restriction was applied. Trials comparing different doses of the same intervention or applying re-randomization of the same sample (i.e. crossover design) and trials that lacked either PSM or a proper comparator arm were excluded. Studies reporting on solitary kidneys or procedures other than CT scans were also excluded. The same applied to patient cohorts undergoing multiple CT scans within 72 h. Systematic reviews and meta-analyses were excluded; however, the references of all identified reviews were searched for additional citations [17–19].

### Study selection and data collection process

The data of selected studies was independently extracted by three investigators (M.O., M.M.G., C.V.B.). In case of discrepancies on study findings, outcomes were discussed (with E.D., supervised by B.M.W.S. and K.W.), and consensus was established. Points of discussion included the handling of articles with mixed CM pathways (i.e. intravenous and intra-arterial), overlapping study populations, differing matching models and the consideration of multiple AKI criteria in the same study.

**Fig. 1:**
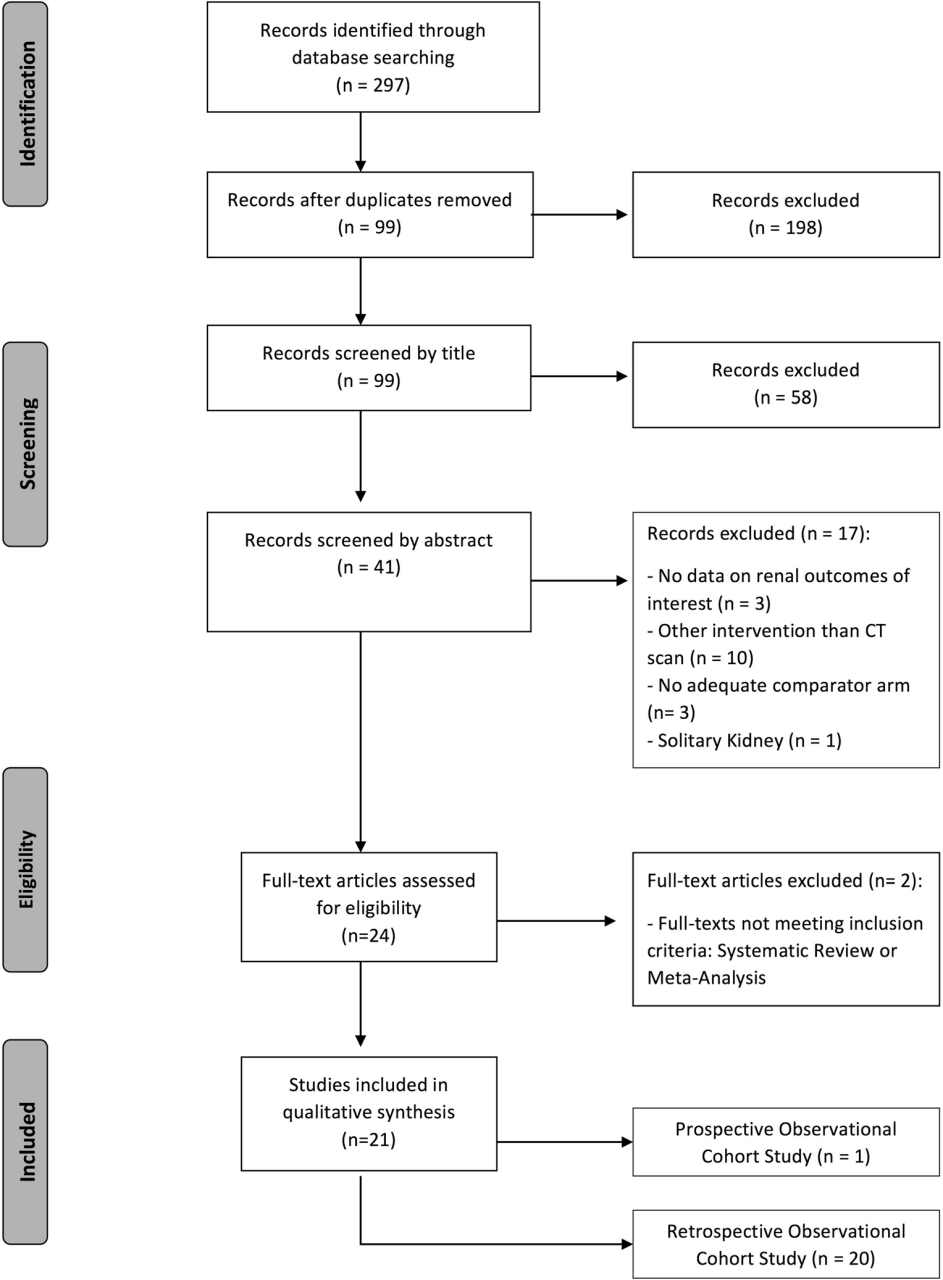
Flowchart showing the study selection process. The numbers of studies identified, assessed for eligibility, and included in the meta-analysis

Our initial search identified 297 publications (Fig. 1). After duplicates were removed, the remaining 99 articles were reviewed by title, leading to the exclusion of 58 records. The remaining 41 articles were screened by abstract. All papers fulfilling the inclusion criteria (*n* = 26) were assessed by full-text review. Potential doubling or reutilization of study populations was thoroughly checked. Ultimately, 21 articles were selected for final data extraction, comprising data of 169,455 patients undergoing CT.

### Data items and statistical analyses

The number of subjects and the study type were retrieved for each study (Table 1). Additionally, the applied matching ratios (i.e. 1:1, 1:3), type of study population (e.g. general population, septic patients) and applied AKI definition (i.e. AKIN, KDIGO, RIFLE or CIN) were identified. The Newcastle–Ottawa Scale was implemented to assess the risk of bias [20]. The calculated scores were converted to the Agency for Healthcare Research and Quality (AHRQ) standards, marking the risk of bias as unclear, high, moderate or low. Cochrane’s *Q* and *I*^2^ were used to indicate the heterogeneity between studies, and a funnel plot was applied to examine the risk of publication bias. A random-effects model (DerSimonian and Laird method) was used to calculate pooled odds ratios (ORs) for primary (i.e. AKI) and secondary outcomes (i.e. dialysis, death) in CM–exposed and unexposed cohorts [21]. Additional meta-regression analysis was performed to determine heterogeneity by patient-related factors (e.g. age, gender, comorbidities).

**Table 1:** Study characteristics and study populations

	Study type	AKI definition	Matching method	Study population	Number of exposed	Number of controls	Odds ratio (OR) [95% confidence interval]
Davenport, 2013a	RCS	AKIN*	1:1	General inpatient population	10,121	10,121	0.96 [0.87 to 1.06]
Davenport, 2013°	RCS	AKIN	1:1	General inpatient population	8826	8826	1.02 [0.91 to 1.15]
Ehrmann, 2013	PCS	CIN^+^	1:1	ICU patients	146	146	1.00 [0.38 to 2.66]
McDonald, 2013	RCS	CIN	1:1	General inpatient population	10,686	10,686	0.94 [0.83 to 1.07]
McDonald, 2015°	RCS	AKIN	1:1	CKD patients	1639	1639	0.78 [0.64 to 0.95]
Hinson, 2016	RCS	AKIN	N/A^^^	ED patients	7201	5499	0.75 [0.66 to 0.85]
Tao, 2017	RCS	AKIN	1:1	Nephrotic syndrome patients	543	543	0.54 [0.32 to 0.91]
Chaudhury, 2018	RCS	CIN	1:1	CKD patients	200	200	1.00 [0.63 to 1.58]
Latcha, 2018	RCS	RIFLE^&^	1:1	Cancer patients	2252	2252	0.98 [0.85 to 1.12]
Ellis, 2019	RCS	AKIN	1:1	Patients with stage IIIb–V CKD	599	599	1.23 [0.91 to 1.67]
Goto, 2019	RCS	KDIGO^§^	1:1	Septic patients	100	100	0.96 [0.54 to 1.71]
Hinson, 2019	RCS	KDIGO	N/A	Septic patients	1464	976	0.75 [0.56 to 1.0]
Puchol, 2019	RCS	AKIN	N/A	ED patients	6642	6193	0.73 [0.64 to 0.83]
Williams, 2019	RCS	KDIGO	1:1	ICU patients	2306	2306	1.09 [0.94 to 1.26]
Gilligan, 2020	RCS	AKIN	1:1	Pediatric patients	925	925	0.92 [0.51 to 1.64]
Elias, 2021	RCS	AKIN	1:1	Patients with suspected pulmonary embolism	969	969	1.00 [0.79 to 1.27]
Guo, 2021	RCS	KDIGO	1:1	Infants and young children undergoing cardiac surgery	159	159	1.09 [0.68 to 1.76]
Gorelik, 2021	RCS	KDIGO	1:1	General inpatient population	11,664	11,664	0.86 [0.78 to 0.95]
Kene, 2021	RCS	AKIN	1:1	Emergency patients with chronic kidney disease	5589	5589	1.68 [1.49 to 1.90]
Su, 2021	RCS	KDIGO	N/A	Emergency patients	10,143	11,921	1.36 [1.25 to 1.49]
Yan, 2021	RCS	AKIN	1:1	Hospitalized acute kidney injury patients	1172	1172	0.86 [0.64 to 1.15]

^*^Acute Kidney Injury Network (AKIN) Definition: Absolute increase of ≥ 0.3 mg from baseline serum creatinine at 48 to 72 hours

^+^Contrast-induced nephropathy (CIN) definition by the European Society of Urogenital Radiology:  absolute SCr increase of 0.5 mg/dL or > 25% of the baseline within 72 h of contrast administration

^§^Kidney Disease: Improving Global Outcomes (KDIGO) definition: absolute SCr increase of ≥ 0.3 mg/dL (26.5 μmol/L) from baseline serum creatinine within 48 h or > 1.5-fold from baseline within 7 days

^&^Risk, Injury, Failure; Loss, End-Stage Renal (RIFLE) definition of AKI: relative increase of 1.5–1.9 over baseline SCr at 48 to 72 h or glomerular filtration rate (GFR) decrease of > 50%

° Studies not included in the main analysis

^ Not applicable

All analyses were performed using Comprehensive Meta-Analysis (CMA, Version 2.2.064) and R 4.0.2.

## Results

The initial literature search identified 297 articles fulfilling our inclusion criteria. After performing full-text article workups, 26 studies were initially declared eligible for inclusion. In the course of our statistical analysis, we detected a high degree of overlapping samples in the studies by Davenport et al and McDonald et al, respectively. In order to address single author bias and increased subject weighting, we decided to remove all duplicates and consider only one study of each author. Based on the largest sample size, the studies from Davenport et al from 2013 [22] and McDonald et al from 2013 [23] were chosen for meta-analysis. As both studies lacked further subdivision into eGFR groups, the 2013 study by Davenport et al [24] and 2015 article by McDonald et al [25] were chosen for the analysis of cohorts with eGFR less than or equal to 30 mL/min/1.73 m^2^.

Ultimately, 21 studies were selected for final data extraction and analysis, six of which consisted of general population cohorts [22–27] (Table 1). Two studies focused on critically ill [28, 29] and two on pediatric patients [30, 31]. Four studies consisted of patients admitted via emergency department [32–35]; one study examined nephrotic syndrome patients [36]; two focused on patients with chronic kidney disease (CKD) [37, 38]; and two on patients hospitalized with AKI [39, 40]. Two groups studied septic [38, 41] and one cancer patients [42]. AKI was defined by RIFLE criteria in 1 study; by AKIN in 11 studies; by KDIGO criteria in 6 studies, and by CIN criteria in 3 studies. If studies applied AKIN and CIN criteria to define AKI, the 2007 definition by AKIN was preferred over CIN. Each study comprised two cohorts that were assigned by PSM. Here, 17 studies (80%) applied a 1:1 matching ratio. Low-osmolar contrast agents were administered in 14 studies (70%), iso-osmolar CM in one study (5%) and a combination of both in five studies (25%). One study (5%) did not specify the type of CM used. The use of high-osmolar CM was declared by none.

### Effects of contrast media administration on kidney function

**Fig. 2:**
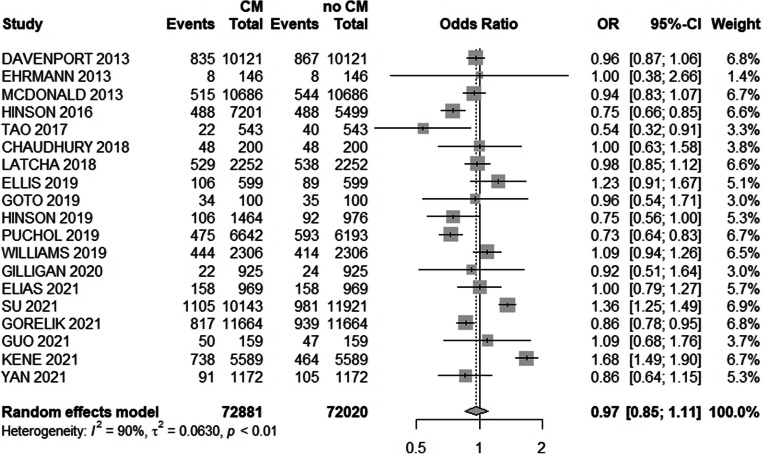
Forest plot with overall odds ratio (OR) of the association of CM application and AKI. 95%-CI, 95% confidence interval; CM, contrast media

Overall, 7425 AKI events were detected in 60,367 patients with CM exposure and 7346 events in 51,980 controls (Table 2). There was a tendency towards lower odds of AKI in CM–exposed cohorts compared with unexposed controls (OR 0.97 [0.85; 1.11], *p* = 0.64) (Fig. 2). Substantial heterogeneity was detected (*I*^2^ = 90.1%, *Q* = 40.74, *p* ≤ 0.0001).

**Table 2:** Acute kidney injury (AKI), dialysis and mortality in exposed and control populations

Study	AKI in exposed	AKI in control	Dialysis in exposed	Dialysis in control	Mortality in exposed	Mortality in control
Davenport, 2013	835	867	N/A	N/A	N/A	N/A
Davenport, 2013a	619	606	N/A	N/A	N/A	N/A
Ehrmann, 2013	8	8	3	4	N/A	N/A
McDonald, 2013	515	544	N/A	N/A	N/A	N/A
McDonald, 2015	215	266	12	8	189	218
Hinson, 2016	488	488	27	49	N/A	N/A
Tao, 2017	22	40	16	21	0	0
Chaudhury, 2018	48	48	N/A	N/A	N/A	N/A
Latcha, 2018	529	538	N/A	N/A	N/A	N/A
Ellis, 2019	106	89	N/A	N/A	N/A	N/A
Goto, 2019	34	35	26	23	17	17
Hinson, 2019	106	92	N/A	N/A	N/A	N/A
Puchol, 2019	475	593	N/A	N/A	N/A	N/A
Williams, 2019	444	414	10	6	N/A	N/A
Gilligan, 2020	22	24	N/A	N/A	N/A	N/A
Elias, 2021	158	158	9	12	N/A	N/A
Guo, 2021	50	47	N/A	N/A	0	0
Gorelik, 2021	817	939	60	45	1000	985
Kene, 2021	738	464	39	17	397	475
Su, 2021	1105	981	500	564	N/A	N/A
Yan, 2021	91	105	27	39	46	56

**Fig. 3:**
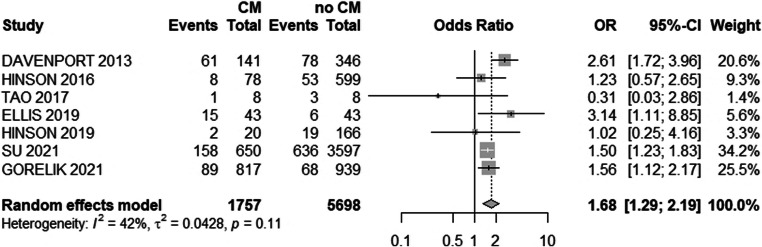
Forest plot with overall odds ratio (OR) of the association of CM application and AKI in patients with eGFR ≤ 30. 95%-CI, 95% confidence interval; CM, contrast media

Since the risk for AKI after CM exposure is presumably higher among patients with impaired kidney function, we performed additional subgroup analysis by aggregating data from patients with eGFR ≤ 30 mL/min/1.73 m^2^ (Fig. 3). Here, we detected a significantly higher risk of AKI in CM–exposed patients compared with unexposed controls (OR: 1.68 [1.29; 2.19], *p* = 0.0001; 55; *I*^2^ = 42%, *Q* = 10.3, *p* = 0.1125) (Fig. 3). Notably, the absolute risk increase in CM–exposed patients with eGFR ≤ 30 mL/min/1.73 m^2^ remains rather low (334/1757, 19% vs. 863/5698, 15%) with an absolute risk increase of 4%.

### Meta-regression analysis

**Fig. 4:**
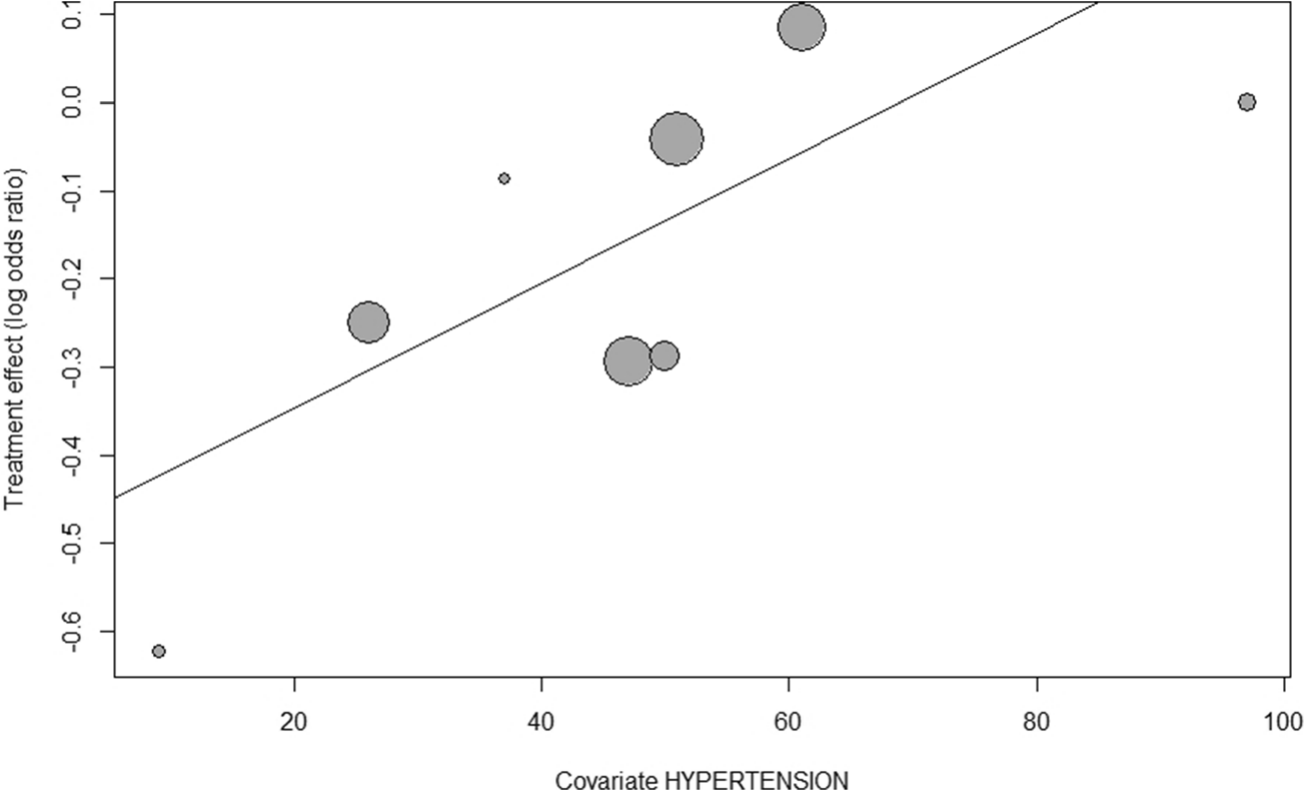
Balloon plot with log odds ratio (OR) of the association of CM application and AKI in patients with hypertension. 95%-CI, 95% confidence interval; CM, contrast media

To further explore possible origins of heterogeneity, we performed meta-regression analyses for 7 relevant covariates (Table 3). Here, a larger proportion of patients with hypertension was associated with higher odds for AKI after CM exposure (*p* = 0.03) (Fig. 4). Other clinically plausible variables related to the use of CM showed no significant influence on AKI rates. Therefore, hypertension and eGFR less than or equal to 30 mL/min/1.73 m^2^ likely are conditions accounting for the observed heterogeneity (Figs. 3 and 4).

**Table 3:** Results of meta-regression (mixed-effects regression)

Covariates	*n* studies	Mixed-effects model		*p* value
		Point estimate	Standard error	
Female gender (%)	11	0.02	0.01	0.15
CHF (%)	11	0.00	0.00	0.88
Diabetes mellitus (%)	10	0.01	0.1	0.2
CKD (%)	11	0.00	0.00	0.88
Hypertension (%)	8	0.01	0.00	0.03
GFR < 60 (%)	9	0.01	0.01	0.31
GFR < 30 (%)	11	0.00	0.01	0.0001

### Effects of contrast media on dialysis and mortality

A total of 1517 patients from 11 studies required renal replacement therapy after CM exposure, including 729 cases from the CM–exposed cohorts (*n* = 18,493) and 788 from the control groups (*n* = 11,202). No significant difference in the rate of dialysis was observed between CM exposed and controls (OR: 0.97 [0.75; 1.25]; *p* = 0.81) (Table 2).

Further, 3400 deaths were reported in 7 studies (*n* = 10,312), including 1649 cases among 8030 CM–exposed patients (13%) and 1751 fatal events in 2282 controls (13%). No significant difference in mortality was detected between the CM groups and controls (OR: 0.94 [0.86; 1.03]; *p* = 0.18) (Table 2).

### Serum creatinine measurements for AKI diagnosis

Our selected studies applied different observation periods to diagnose AKI after CT imaging. Here, SCr levels were acquired within 24 h [22, 24, 29], 48 h [22, 24, 30, 31], 72 h [22, 24, 26, 42] or 96 h after the index CT scan [28, 37]. In three studies, the window for post-contrast SCr measurement reached from 48 to 72 h [33, 34, 41], one of which added a second time point between 48 h and 1 week. One group measured the SCr levels for 1 month after index CT [40], while one study refrained from defining the time period [39].

The frequency of SCr measurements following contrast-based imaging differed markedly among our studies. While three groups obtained only one SCr level after imaging [32, 33, 41], two studies measured at least three early SCr values in each 24-h period for the first 72 h after index CT [22, 24]. Eight groups reported more than one SCr measurement without disclosing the exact number [24, 26, 32, 39, 43–46]. For the remaining studies, the number of measurements was not disclosed [23, 25, 27, 30, 31, 34, 35, 38].

### Risk of bias within studies

The Newcastle–Ottawa Scale indicated a low risk of bias for all studies according to the AHRQ standard (Table 4). The funnel plot (Suppl. Fig. 1) revealed no evidence of relevant publication bias. Because we hypothesized that differences in propensity score–matching methods might introduce between-study heterogeneity, an additional meta-regression analysis was performed to verify the adequacy of matching procedures (Suppl. Fig. 2). Here, we found that the inclusion of more variables in the matching model (range: 3 to 42) was associated with a greater tendency towards similar incidences of AKI between exposed and unexposed groups (*p* = 0.093).

**Table 4:** Risk of bias assessment with the Newcastle–Ottawa Scale

	Selection				Comparability	Outcome		
Study and Year	Representative Cohort	Selection of Non-Exposed	Ascertainment of exposure	Outcome not present at outset	Comparability	Outcome assessment	Duration Follow-Up	Adequacy of Follow-Up
Davenport, 2013	*	*	*	*	**	*	*	*
Davenport, 2013a	*	*	*	*	**	*	*	*
Ehrmann, 2013		*	*	*	**	*	*	*
Mcdonald, 2013	*	*	*	*	**	*	*	*
Mcdonald, 2015		*	*		**	*	*	*
Hinson, 2016		*	*	*	**	*	*	*
Tao, 2017		*	*		**	*	*	*
Chaudhury, 2018	*	*	*		**	*	*	*
Latcha, 2018	*	*	*	*	**	*	*	*
Ellis, 2019		*	*		**	*	*	*
Goto, 2019	*	*	*		**	*	*	*
Hinson, 2019	*	*	*		**	*	*	*
Puchol, 2019	*	*	*	*	**	*	*	*
Williams, 2019	*	*	*		**	*	*	*
Gilligan, 2020	*	*	*	*	**	*	*	*
Elias, 2021	*	*	*	*	**	*	*	*
Guo, 2021		*	*	*	**	*	*	*
Gorelik, 2021	**	*	*	*	**	*	*	*
Kene, 2021		*	*		**	*	*	*
Su, 2021	*	*	*	*	**	*	*	*
Yan, 2021		*	*		**		*	*

The Newcastle–Ottawa Scales were used to assess the risk of bias for the cohort studies. Each domain was rated on a scale of zero or one star, except comparability, which can be awarded up to two stars. 0 = High or unclear risk of bias; 1 or 2 = Low risk of bias

## Discussion

For years, the literature has been shaped by the assumption that post-contrast AKI is attributable to the iodinated CM itself rather than preexisting nephrotoxic risk factors. Building on prior studies, we sought to facilitate clinical decision-making and prevent both over- and underestimation of AKI risk during CT examination. Risk overestimation might deprive patients of clinical benefits of contrast-enhanced imaging out of fear of causing AKI. However, an underestimation could expose patients to preventable nephrotoxic insults with high potential for adverse outcomes.

We performed a systematic review and meta-analysis of 21 cohort studies utilizing a propensity-matched multivariate model, in order to isolate the role of CM exposure on the incidence of post-contrast AKI. In line with the growing body of literature, we found no evidence from state-of-the-art cohort studies for an increased risk for AKI, dialysis or mortality after single administration of CM during CT scan in eGFR groups above 45 mL/min/1.73 m^2^. These results appear robust, even in subgroups with chronic and critical illness. However, our analysis revealed an increased risk of AKI in patients with eGFR of less than or equal to 30 mL/min/1.73 m^2^ and hypertensive disease.

Previous studies on CM nephrotoxicity were limited either by a lack of control groups or by absence of adjustments for predisposing risk factors [43, 44, 47, 48]. The study by Moos et al [48] has stood out by including four studies with unexposed controls (out of a total of 41 studies). Retrospective observational studies followed, showing similar rates of AKI following CT examination regardless of CM administration [45, 46]. Moreover, a substantial number of patients without CM exposure displayed changes in SCr levels that would have met the criteria for CIN, had they undergone CM administration [45]. This emphasized the need for a proper comparator arm. Observational controlled studies followed, presenting similar rates of AKI between CM–exposed patients and unexposed controls. Currently, PSM protocols are employed to adjust for patient-related factors (e.g. age, sex) and various underlying comorbidities amongst study cohorts, thereby approximating a randomized distribution. Our study further expands upon contemporary meta-analyses that either partly [49] or entirely [50] lacked matched controls, or featured considerably fewer studies [19].

Our findings reverberate the conflicting data in the adult literature regarding renal risks after intravenous CM administration and prompt a differential analysis for patients with high disease burden.

Assessing a broad range of comorbidities, no other association with higher AKI risk was found. This is particularly noteworthy considering that our study populations featured critically [28, 29, 34, 35] and chronically ill patients [25, 37, 39]. In daily clinical practice, these patients are most likely to experience exclusion from CM–enhanced procedures out of fear of causing contrast-induced AKI. Our findings do not support the clinical avoidance of CM where otherwise indicated. Similarly, van der Molen et al recently demonstrated no need for emergency haemodialysis after administration of iodine-based CM in patients with dialysis-requiring CKD [51]. The observed shift in the medical literature may be explained by adjustments in CM osmolality and administered volumes in recent years.

Similar to other authors, we observed a trend towards lower risk for renal impairment after CM exposure [32, 33]. Puchol et al explained this with the hydration occurring in the course of administering the CM volume, and its subsequent osmotic diuretic effects [32]. Since CM is not nephroprotective, we assume the presence of factors affecting the AKI incidence. These are not easily measurable and, as it seems, not entirely rectified by PSM. Investigators who applied PSM models have reported similar results [52]. Studies cannot consider factors that conceivably bias the decision to administer CM in the first place. Therefore, it remains crucial to consider the possible impact of these variables before and after PSM, in order to avoid misleading inferences of causality. Selection bias may also cause the higher number of AKI among controls (i.e. unenhanced CT). This arises when presumed high-risk patients are excluded from CM exposure under the assumption that CM causes AKI, which precipitates a less healthy control cohort. Likewise, discrepancies in matching methodologies or small sample sizes may contribute to this finding [22]. Conceivably, patients with contrast-enhanced imaging might receive a better fluid management as part of the CM administration protocol.

Our study displays various methodological strengths. We focused on cohort studies originally designed to compare the nephrotoxicity of CM–enhanced CT with unenhanced CT examinations. By restricting the analysis to studies that applied PSM, we further narrowed limitations of inherent biases in observational study designs [53]. Our findings affirm that PSM does not account for all influencing factors and that all outcomes require careful interpretation. However, since randomized controlled trials evaluating post-contrast AKI remain unlikely for ethical reasons, our findings summarize the best available evidence in absence of randomization. By excluding all studies that lacked controls, we further enhanced the rigor of our analysis. AKI diagnosis was made based on internationally recognized guidelines, the anticipated primary event of interest was documented and standardization across all studies was established in terms of design (i.e. observational cohort study), intervention (i.e. CT scan) and primary outcome (i.e. AKI).

To the best of our knowledge, we provide the first and most extensive study that systematically assesses the renal risks after CT examination attributable to CM after controlling for demographic variables. Despite this, we note limitations in our study, which deserve mention. Our data relies on retrospective cohort studies with limited numbers of participants. One study did not disclose the osmolality of CM used. However, based on the recency of publication (i.e. 2019), the use of high-osmolar CM for CT is quite unlikely. Further, neither fluid administration during and after CT nor nephrotoxic medications were consistently documented throughout the studies. This would have been preferable, as hydration is known to reduce the risk of post-contrast renal impairment [51]. With regard to total volumes of injected CM, only weight-adjusted ranges were provided (*n* = 15). Notably, none of the groups described the flow rate of intravenous CM administration and only eight disclosed CM concentrations. We strongly recommend the disclosure of all periprocedural circumstances for accurate risk estimation and effective periprocedural management.

This also applies to post-contrast serial measurements of SCr in clinical settings. Since AKI is not necessarily associated with permanent changes in renal function, consistent SCr measurement protocols following CM administration would be of great value to improve the diagnostic algorithm in suspected AKI. Diagnostic standardization with longer observation periods may help differentiate between subclinical renal damage and potentially reversible background fluctuations of SCr. Lastly, the majority of studies failed to report AKI stages, which would have been beneficial to understand the severity of kidney injury and show the risk of progression to higher AKI stages.

Currently, no adjunctive medication can effectively prevent or treat post-contrast AKI. Therefore, it remains crucial to anticipate and obviate post-contrast renal decline with comprehensive risk prediction scores and preprocedural volume expansion, even in emergencies and time-sensitive conditions [54]. The clinical practice of withholding CM–enhanced imaging for concern of CI-AKI appears not to be justified. However, despite the low incremental risk, caution remains warranted in individuals with hypertension or eGFR less than or equal to 30 mL/min/1.73 m^2^.

## Supplementary Information

ESM 1 (PDF 627 kb)

## Abbreviations

AKI: Acute kidney injury
AKIN: Acute Kidney Injury Network
CA-AKI: Contrast-associated acute kidney injury
CI-AKI: Contrast-induced acute kidney injury
CIN: Contrast-induced nephropathy
CM: Contrast media
SCr: Serum creatinine
CT: Computed tomography
KDIGO: Kidney Disease: Improving Global Outcomes
RIFLE: Risk, injury, failure, loss of kidney function, and end-stage kidney disease
95%CI: 95% confidence interval
PS: Propensity score
PSM: Propensity score matching
CKD: Chronic kidney disease

## Declarations

### Guarantor

The scientific guarantor of this publication is Professor Bernhard Magnus Wilhelm Schmidt, MD, SM/M.Sc.

### Statistics and biometry

One of the authors has significant statistical expertise.

### Methodology

• retrospective

• not applicable

• performed at one institution
